# Supplementary material for: Acceptance and compliance with micronutrient powder (MNP) among children aged 6–23 months in northern Nigeria
Source: PLOS Glob Public Health. 2022 Oct 17;2(10):e0000961. doi: 10.1371/journal.pgph.0000961 (PMC10022258; doi:10.1371/journal.pgph.0000961)
Supplement: S1 Dataset — (ZIP) [file pgph.0000961.s008.zip › S8_Dataset/Spot Checks.zip!/Spot Checks/04072015_A_SO_PM_2_SC.b.pdf]

SPOT CHECK RECORDING FORM

State: Adamawa LGA: ____Song____ Community: _Galamba _

Name of caregiver (female): Salama Abednego

Name or caregiver (male): ………………………

Sex of index child: _male___ Age of index child (in months): _17 months_____________

Spot Check Information

1. **Date of MNP distribution to household** (circle option below based on LGA name above)**:**
   - **Kebbi:** BirninKebbi&Jega LGA: **June 16**
   - **Kebbi:** Danko-wasagu: **June 17**
   - **Adamawa:**Song, Ganye: **(June 18)**
   - **Adamawa:**Guyuk **June 19**
2. **Date of Spot Check Observation: ____4/7/2015_____**
3. **Number of sachets remaining**(Ask the caregiver to show you and count the specific number of sachets remaining)**:**

   42

4. **Number of sachets that should remainusing one MNP sachet per day** (Analyst only)**:**

   45

5. **Number of sachets remaining / number of sachets should be remaining**(Analyst only)**:**

_____42____ / ___45______ = __93.3__ %

Probes

Question 1. You have **XX** MNP sachets remaining. Explain how easy or difficult it was to use the MNP product on a daily basis for your child 6 – 23 months? (Circle one answer)

Very Difficult **Difficult** **(Easy)** **(Very Easy)**

Question 2. Describe any specific challenges that you faced that made the MNP difficult to use on a daily basis?

- Increase in food consumption.
- The boy looks more heavy in weight
- I always have the feeling that my child is consuming a drug daily that will make him stay healthy always

Question 3. Describe specific aspects of the MNP product that you or your child enjoyed that made it easier to use on a daily basis?

- What is the benefits of this MNP in the body of the child.
- Would this MNP be sold or distributed free

Question 4. What questions do you have about the MNP based on your experience using it?

NIL

Question 5. What specific recommendations do you have that we can do as a program to make the MNP easier to use on a daily basis?

NIL
